# Fasudil and viscosity of gelatin promote hepatic differentiation by regulating organelles in human umbilical cord matrix-mesenchymal stem cells

**DOI:** 10.1186/s13287-024-03851-9

**Published:** 2024-07-29

**Authors:** Jiwan Choi, Seoon Kang, Hye-In An, Chae-Eun Kim, Sanghwa Lee, Chan-Gi Pack, Young-In Yoon, Hana Jin, Yong-Pil Cho, Chong Jai Kim, Jung-Man Namgoong, Jun Ki Kim, Eunyoung Tak

**Affiliations:** 1 Department of Convergence Medicine, Asan Medical Center, University of Ulsan College of Medicine, Seoul, Republic of Korea; 2 Asan Medical Institute of Convergence Science and Technology (AMIST), Asan Medical Center, University of Ulsan College of Medicine, Seoul, Republic of Korea; 3 Biomedical Engineering Research Center, Asan Medical Center, Seoul, Republic of Korea; 4 Department of Biomedical Engineering, College of Medicine, University of Ulsan, Seoul, Republic of Korea; 5 Division of Hepatobiliary Surgery and Liver Transplantation, Department of Surgery, Asan Medical Center, University of Ulsan College of Medicine, Seoul, Republic of Korea; 6 Division of Vascular Surgery, Department of Surgery, Asan Medical Center, University of Ulsan College of Medicine, Seoul, Republic of Korea; 7 Department of Pathology, Asan Medical Center, Asan-Minnesota Institute for Innovating Transplantation (AMIT), University of Ulsan College of Medicine, Seoul, Korea; 8 Department of Pediatric Surgery, Asan Medical Center Children’s Hospital, University of Ulsan College of Medicine, Seoul, Republic of Korea

**Keywords:** Human umbilical cord matrix-mesenchymal stem cells, Gelatin viscosity, ROCK inhibitor, Fasudil, Hepatic differentiation, Mitochondria activation, Lipid droplet, Stem cell organelles

## Abstract

**Background:**

Human mesenchymal stem cells originating from umbilical cord matrix are a promising therapeutic resource, and their differentiated cells are spotlighted as a tissue regeneration treatment. However, there are limitations to the medical use of differentiated cells from human umbilical cord matrix-mesenchymal stem cells (hUCM-MSCs), such as efficient differentiation methods.

**Methods:**

To effectively differentiate hUCM-MSCs into hepatocyte-like cells (HLCs), we used the ROCK inhibitor, fasudil, which is known to induce endoderm formation, and gelatin, which provides extracellular matrix to the differentiated cells. To estimate a differentiation efficiency of early stage according to combination of gelatin and fasudil, transcription analysis was conducted. Moreover, to demonstrate that organelle states affect differentiation, we performed transcription, tomographic, and mitochondrial function analysis at each stage of hepatic differentiation. Finally, we evaluated hepatocyte function based on the expression of mRNA and protein, secretion of albumin, and activity of CYP3A4 in mature HLCs.

**Results:**

Fasudil induced endoderm-related genes (*GATA4, SOX17*, and *FOXA2*) in hUCM-MSCs, and it also induced lipid droplets (LDs) inside the differentiated cells. However, the excessive induction of LDs caused by fasudil inhibited mitochondrial function and prevented differentiation into hepatoblasts. To prevent the excessive LDs formation, we used gelatin as a coating material. When hUCM-MSCs were induced into hepatoblasts with fasudil on high-viscosity (1%) gelatin-coated dishes, hepatoblast-related genes (*AFP* and *HNF4A*) showed significant upregulation on high-viscosity gelatin-coated dishes compared to those treated with low-viscosity (0.1%) gelatin. Moreover, other germline cell fates, such as ectoderm and mesoderm, were repressed under these conditions. In addition, LDs abundance was also reduced, whereas mitochondrial function was increased. On the other hand, unlike early stage of the differentiation, low viscosity gelatin was more effective in generating mature HLCs. In this condition, the accumulation of LDs was inhibited in the cells, and mitochondria were activated. Consequently, HLCs originated from hUCM-MSCs were genetically and functionally more matured in low-viscosity gelatin.

**Conclusions:**

This study demonstrated an effective method for differentiating hUCM-MSCs into hepatic cells using fasudil and gelatin of varying viscosities. Moreover, we suggest that efficient hepatic differentiation and the function of hepatic cells differentiated from hUCM-MSCs depend not only on genetic changes but also on the regulation of organelle states.

**Supplementary Information:**

The online version contains supplementary material available at 10.1186/s13287-024-03851-9.

## Background

Mesenchymal stem cells (MSCs) originate from human fetal and adult tissues, such as bone marrow, umbilical cord matrix, placenta, and various adult tissues [1]. Specifically, two fetus-originated tissues, umbilical cord matrix and placenta, are excellent sources of human MSCs because of their prominent advantages, such as a painless collection procedure, faster self-renewal and the ability of differentiation into three germ layers [2]. Thus, recently, human umbilical cord matrix-derived MSCs (hUCM-MSCs) and their differentiated cells have been used for tissue regeneration in therapeutic medicine to treat various diseases [3].

The liver, essential for drug detoxification and biosynthesis of proteins and hormones, cannot be easily substituted by other organs. Consequently, many patients remain on transplant waiting lists, as healthy liver cells are the sole replacement for irreversibly damaged hepatic cells [4, 5]. Therefore, advanced stem cell technologies can provide great hope for patients facing end-stage diseases with no alternative but organ transplantation [6]. However, there are restrictions on the medical use of differentiated cells from stem cells in therapies. One of the biggest obstacles to the use of these cells is the efficiency of differentiation protocols and the limited phenotypes of mature cells [7]. For example, hepatocyte-like cells derived from human MSCs using current protocols exhibit characteristics more similar to fetal hepatocytes than the adult cells in terms of transcriptome profiles, hepatic functions, and metabolic activities [8]. Thus, numerous differentiation methods have been developed, including approaches using genetic modifications, microenvironment adjustments, and the addition of cytokines and growth factors [9].

In stem cell fate determinations, transcriptome changes are strongly linked to the differentiation of cell types. Therefore, recently, the transcription of differentiated cells has been world widely analyzed using sequencing tools, such as RNA sequencing [10]. However, cell fate does not only change transcriptional regulation. Cellular differentiation and lineage commitment are affected by communication between nuclei and various biological processes and signaling pathways involving cytoplasmic macromolecule and organelle interactions [11–13]. In particular, changes of metabolism are accompanied when stem cells are differentiated, and it is known to play a vital role in stem cell fate determinations [14]. In cell metabolism, mitochondrial dynamics are pivotal in determining cell fate and function [15]. Moreover, it has been reported that lipid droplets (LDs), which are related to storage organelles at the center of lipid and energy homeostasis, are also linked with stem cell fate determination [16]. Although the states of stem cell organelles are important for determining stem cell fate, it is still largely unknown whether the regulation of stem cell organelle states affects hepatic differentiation.

This study demonstrated that the hepatic differentiation of hUCM-MSCs is significantly influenced not only by transcriptomic alterations but also by the state of organelles. We found that fasudil induced endoderm genes in the early differentiation, but facilitated the excessive accumulation of LDs in stem cells and interfered with hepatic differentiation. However, when hUCM-MSCs were reacted with fasudil in a high-viscosity gelatin-coated dish reduced the accumulation of LDs, activated mitochondrial function, and increased the efficiency and function of differentiated cells. Moreover, in the mature stage of differentiation, low-viscosity gelatin reduced the induction of LDs and activated mitochondria, thereby increasing the differentiation efficiency and function of differentiated cells. Collectively, our study findings highlight the importance of the hepatic differentiation of hUCM–MSCs not only to transcriptome changes but also to the regulation of the organelle states of differentiated cells.

## Materials and methods

### Cell culture

In this study, hUCM-MSCs were obtained from the Asan Stem Cell Center (Asan Institute for Life Sciences, Seoul, Korea) [17]. The stem cells were cultured on 0.1% gelatin-coated cell culture dishes in DMEM/F12 medium, supplemented with 10% FBS (fetal bovine serum; GenDEPOT, TX, USA), 1% NEAA (non-essential amino acids), 1% antibiotic–antimycotic (Gibco, USA) and 0.2 mM L-ascorbic acid. Each manually passaged at 1:3 to 1:5 dilutions every 3–4 days.

### Quantitative RT-PCR

Total RNA was extracted using an RNeasy Mini Kit (Qiagen, CA, USA) following the manufacturer’s instructions. Complementary DNA (cDNA) was synthesized using an Ultrascript 2.0 cDNA Synthesis Kit (PCR Biosystems, London, UK), and qRT-PCR was performed using HOT FIREPol EvaGreen qPCR Supermix (SOLIS BIODYNE, Tartu, Estonia) on a CFX Connect Real-Time PCR Detection System (Bio-Rad Laboratories, Hercules, CA, USA). The mRNA levels were normalized to GAPDH for analysis. The primer sequences are listed in Table S1.

RT-qPCR was conducted to evaluate the expression of hepatic mature miRNAs (miR-122, and miR-192) in undifferentiated and differentiated cells. Briefly, cDNA was synthesized from total RNA using the miRCURY LNA RT Kit (Qiagen, Hilden, Germany) according to the manufacturer’s instructions. RT-qPCR analysis was performed using the miRCURY LNA SYBR Green PCR kit (Qiagen) with microRNA-specific primers purchased from Qiagen. Cycling conditions were as follows: incubation at 95 °C for 2 min, followed by 40 cycles of denaturation for 10 s at 95 °C, and annealing and extension for 1 min at 56 °C. The cycle threshold values were determined using Bio-Rad CFX Maestro software (CFX Maestro, version 1.1; Bio-Rad Laboratories). All experiments were repeated three times, and *RNU6B* was used as an internal control.

### Mitochondrial DNA copy number estimation

Mitochondrial DNA (mtDNA) copy number was determined using the Absolute Human Mitochondrial DNA Copy Number Quantification qPCR assay kit (ScienCell, CA, USA). Before assessing the mtDNA copy number, we isolated total DNA using the QIAamp DNA Mini Kit (Qiagen). Briefly, the cycle threshold values were measured in triplicate for each sample using nuclear-specific and mitochondria-specific probes. The assay was performed according to the manufacturer’s instructions.

### Detection of secreted human albumin

The secreted human albumin from the differentiated cells was detected using a Human Albumin ELISA kit (Bethyl Laboratories, TX, USA) according to the manufacturer’s instructions. Albumin secretion was normalized to the culture day and total cell number.

### Measurement of CYP3A4 activity in vitro

Enzyme activity was determined using the P450-Glo CYP3A4 kit (Promega, WA, USA) according to the manufacturer’s instructions. Luminescence was measured by GloMax 96 Microplate Luminometer (Promega). CYP3A4 activity was normalized to the culture day and double-stranded DNA content of each sample.

### In vitro hepatic differentiation

Hepatic differentiation was performed as previously reported with slight modifications [17, 18]. Briefly, the stem cells were seeded on cell culture dishes at 7000 cells/cm^2^. Cell culture dishes were not coated when evaluating the effect of three different ROCK inhibitors, such as fasudil (AdooQ Bioscience, CA, USA), Y-27632, and ripasudil (Sigma Aldrich, USA), on hUCM-MSCs. On the other hand, in all other experiments, culture dishes were coated with 0.1% or 1% gelatin or 1% Matrigel (Corning, NY, USA) or 1 × vitronectin (Thermo Fisher Scientific, MA, USA). One day after subculture, ROCK inhibitor was treated according to concentration and time for induction of an endoderm.

Next, the cells were cultured in a hepatoblast induction medium consisting of step-1 basal medium, 10 ng/ml FGF2, 20 ng/ml BMP4 (Peprotech, NJ, USA), and 3 μM CHIR99021 (Tocris, UK) for 4 days. Finally, the differentiated cells were cultured in a hepatocyte-like cells induction medium consisting of a step-2 basal medium and 20 ng/ml oncostatin M (OSM, Peprotech) for 8 days. Thereafter, the medium was replaced with a hepatic maturation medium consisting of step-2 basal medium, 20 ng/ml OSM only or OSM with extracellular matrix − 0.1% gelatin or 0.1% Matrigel or 0.1 × vitronectin−for 5 days. Step-1 and step-2 basal medium are composed as described below.

The step-1 basal medium consisted of the following steps: IMDM (Iscove’s Modified Dulbecco’s Medium; Gibco) supplemented with 0.1% PVA (polyvinyl alcohol; Sigma Aldrich), 10 mM nicotinamide (Sigma Aldrich), 20 ng/ml hHGF (human hepatocyte growth factor; PeproTech), 1% ITS (insulin–transferrin–selenium; Gibco), and 1% penicillin/streptomycin (GeneDireX, Taiwan); The step-2 basal medium consisted of the following steps: IMDM supplemented with 1 μM dexamethasone, 1% ITS, 20 ng/ml hHGF, and 1% penicillin/streptomycin.

A protocol of conventional hepatic differentiation was conducted using previously reported differentiation method [19].

### Protein extraction and western blotting

For western blotting, cells were trypsinized, washed with ice-cold PBS, and lysed in RIPA lysis buffer (50 mM HEPES pH 7.4, 150 mM NaCl, 1 mM EDTA, 2.5 mM EGTA, 1 mM DTT, 1% Triton X-100) containing a protease and phosphatase inhibitor cocktail (Sigma Aldrich). After lysis, cell debris was removed by centrifugation at 13,000 rpm for 20 min. The protein concentrations were determined using the Bradford assay. Total cellular proteins (15 µg) were separated by 8–15% SDS–PAGE and transferred to Immobilon PVDF membranes (Millipore, MA, USA). The membranes were blocked with 8% BSA (bovine serum albumin; GenDEPOT) in TBST (Tris-buffered saline with Tween 20; 20 mM Tris–HCl pH 7.4, 150 mM NaCl, 0.1% Tween 20) and probed with anti-Albumin (Abcam, Cambridge, UK) and anti-CYP3A4 (Santa Cruz, CA, USA) primary antibodies. After washing with TBST, the primary antibodies were detected using horseradish peroxidase-conjugated anti-mouse secondary antibodies and an enhanced chemiluminescence detection system (Amersham, Buckinghamshire, UK). Full-length western blotting images are presented in Fig. S5.

### Organelle analysis in the differentiated cells

Label-free optical diffraction tomography (ODT) using refractive index (RI) tomography was conducted on hUCM-MSCs using an ODT microscope (HT-X1; Tomocube Inc., Daejeon, Korea). The ODT used three-dimensional RI tomography to reconstruct a single hUCM-MSCs from 48 overlapping two-dimensional holograms captured at various angles, illuminated by a 450-nm LED (light-emitting diode) in a controlled atmosphere of 5% CO_2_ at 37 °C. The HT-X1 microscope, incorporating a Mach–Zehnder interferometer, was utilized for the three-dimensional RI tomographic reconstruction of the cells. LD quantification and volumetric analysis were performed using TomoAnalysis software by TomoCube. Fluorescent staining was employed to ensure precision. MitoTracker dyes (Invitrogen, CA, USA, 250 nM) for mitochondrial labeling and Biotium LipidSpot 488 lipid droplet stain (1:1000 dilution) were used to stain the mitochondria and LDs, respectively. Live cell staining was performed according to the manufacturers’ instructions.

We also observed the cells using a Zeiss LSM 880 confocal laser scanning microscope (Carl Zeiss, Oberkochen, Germany). The cells were fixed with 4% formaldehyde overnight, washed with the PBST (PBS with Tween 20), permeabilized in 0.5% Triton X-100, and blocked with PBST containing 1% BSA. The samples were stained with Biotium LipidSpot 488 lipid droplet stain. Nuclei were counterstained with NucBlue Fixed Cell ReadyProbes Reagent (DAPI; Invitrogen) for 10 min, and fluorescence signals were detected using the Zeiss LSM 880 confocal laser scanning microscope.

### Seahorse assay

To measure oxygen consumption rate (OCR) in differentiated cells, stem cells were seeded at 7000 cells/cm^2^ in 0.1% or 1% gelatin-coated XFe24 cell culture plates (Agilent Technologies, Santa Clara, CA, USA) and subsequently induced to differentiate. Mitochondrial OCR was measured using an XF Cell MitoStress test kit in an XF24 extracellular flux analyzer (Agilent Technologies). OCR values were normalized by the amount of cellular DNA.

#### Measuring the proliferation capacity during hepatic differentiation

To measure the cell growth rate during hepatic differentiation, stem cells were seeded at 6 × 10^4^ cells in 25T flask. On day 3, 7, and 20 of differentiation, the cells were detached using trypsin, and cell number were counted by hemocytometer. The proliferation capacity was calculated using the formula described follow: day 0 or 3 or 7 or 20 cell number/6 × 10^4^ cells.

#### Statistical analysis

Statistical analysis was performed using GraphPad Prism, version 6.0 (GraphPad Software, MA, USA). Comparisons of three or more data sets were performed by one-way or two-way ANOVA (analysis of variance) followed by Bonferroni’s multiple comparison tests. Two-group comparisons were performed using two-tailed Student’s t-tests. *P* values < 0.05 were considered statistically significant.

## Results

### State of organelles in differentiated cells influence the early stage of hepatic differentiation

**Fig. 1:**
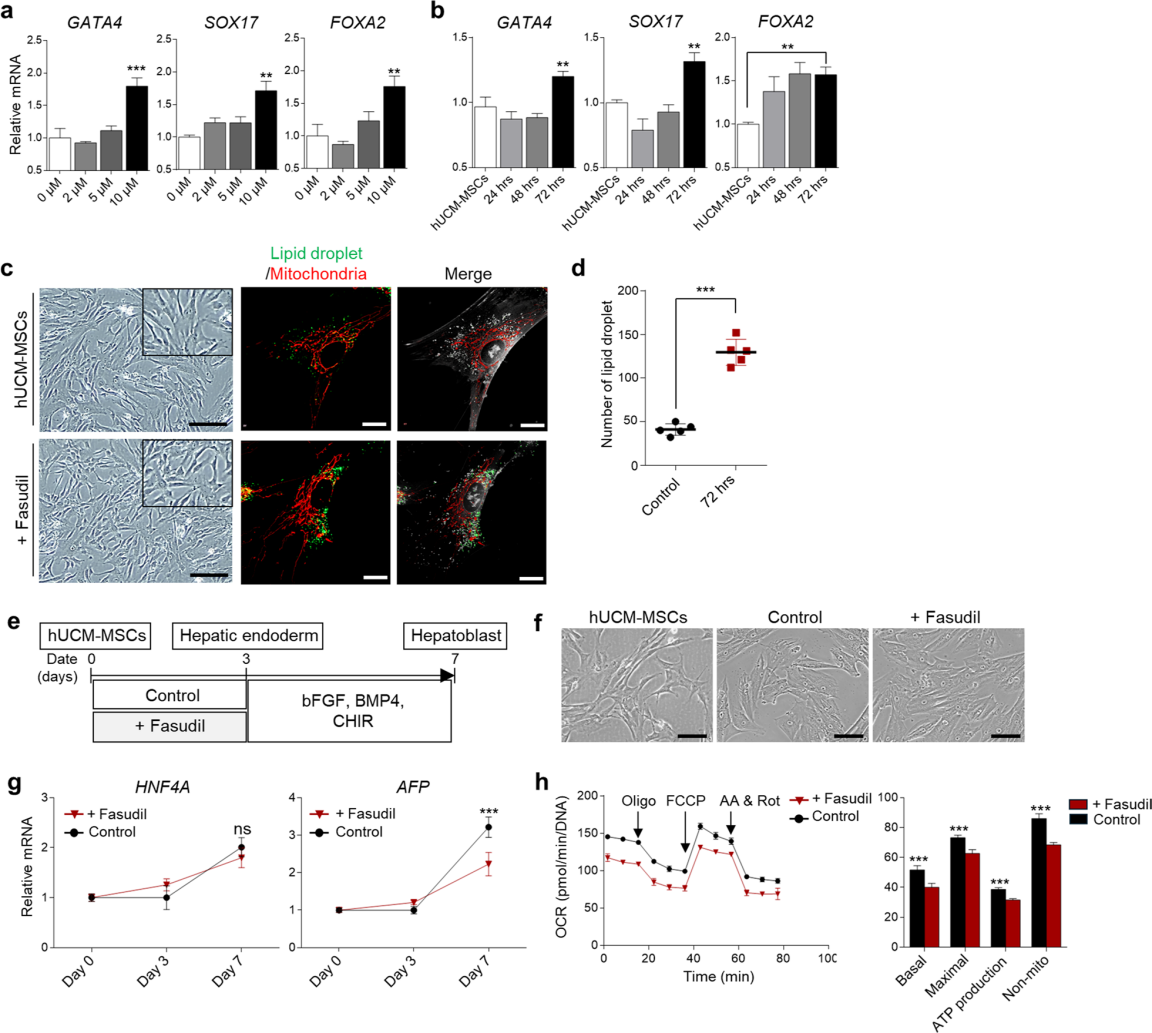
Effect of fasudil on early hepatic differentiation of hUCM-MSCs. **a, b** Transcription analysis of endodermal markers (*GATA4*, *SOX17* and *FOXA2*) expression in cells treated with fasudil according to concentration (a) and time (b). **c** Microscopic and tomographic analysis of the fasudil non-treated and treated group. Mitochondria (red) and lipid droplets (green) were stained with MitoTracker and Biotium LipidSpot, respectively. Scale bar: microscopic, 200 μm; tomographic, 10 μm. **d** The number of lipid droplets in the cells (Control, n = 5 cells; 72 h, n = 5 cells). **e** Experimental scheme of fasudil treatment in hUCM–MSCs for induction of hepatoblast. CHIR: CHIR99021. **f** Morphology of the cells at differentiation day 7. Scale bar = 100 μm. **g** RT-qPCR analysis of hepatoblast markers *AFP* and *HNF4A* on differentiation day 7. GAPDH was used as an internal control for RT-qPCR. **h** Time-dependent oxygen consumption rates (OCR) graph and bar charts for each group on differentiation day 3. Olig: oligomycin, FCCP: carbonyl cyanide 4-(trifluoromethoxy) phenylhydrazone, AA: antimycin A, Rot: rotenone. OCR values were normalized by DNA concentration. Non-mito, non-mitochondrial consumption rate. *P* values < 0.05 were considered significant. *, *P* < 0.05; **, *P* < 0.01; ***, *P* < 0.001

In our initial work, we tested how the ROCK inhibitor, fasudil, influences the differentiation of hUCM–MSCs into hepatic endoderm, a crucial process in the initial stages of cellular differentiation. Previous studies were reported that ROCK inhibitors not only improve stem cell viability but also promote the induction of human pluripotent stem cells (hPSC) into the endoderm [20]. Moreover, differentiation efficiency increased when small molecules were used rather than only protein-used [21]. Thus, in order to confirm the effect of fasudil on the hepatic differentiation of hUCM-MSCs, we first verified a proper concentration and treatment time of fasudil. Unlike the previously reported concentration of fasudil used in hPSCs [22], endoderm markers (*GATA4, SOX17* and *FOXA2*) were significantly increased at a high concentration of 10 μM, and it was effective when treated for 72 h (*P* < 0.05, Fig. 1a, b). We also tested whether other types of ROCK inhibitors, such as Y-27632 and ripasudil, upregulated endodermal genes in hUCM-MSCs. The result showed that all endodermal genes significantly upregulated at a concentration of 10 μM when different types of ROCK inhibitor were used except Y-27632 (*P* < 0.05, Fig. S1a). Among the three different ROCK inhibitors, fasudil more upregulated endodermal genes, except *GATA4*, than two other ROCK inhibitors on differentiation day 3 (*P* < 0.05, Fig. S1b). Next, we conducted tomographic analysis to confirm the state of organelles, such as LDs and mitochondria, within differentiated cells, which were known to be important for metabolism and regulation of stem cell fate [16, 23]. As a result, the stem cells changed to a more ovoid shape and the mitochondrial morphology was different (Fig. 1c). Moreover, LDs were induced inside the cells, and the number of LDs increased (*P* < 0.001, Fig. 1c, d).

Next, we examined whether the use of fasudil increased the differentiation efficiency of hepatoblasts, as an evaluation of the next step in the hepatic endoderm (Fig. 1e). The phenotype of differentiated cells was similar in both the control and fasudil-treated groups (Fig. 1f). However, the expression of hepatoblast-related genes was similar (*HNF4A*, no significant) or lower (*AFP*, *P* < *0.001*) in the fasudil-treated group than the control group at differentiation day 7 (Fig. 1g). To explain the hepatoblast differentiation efficiency decreased despite endodermal gene upregulation, we performed mitochondrial function test. Mitochondrial functions, such as adenosine triphosphate (ATP) production, are related to the efficiency of differentiation and are reduced when excessive LDs are induced [24]. Therefore, we hypothesized that LDs induced by fasudil would affect mitochondrial function. As we expected, mitochondrial function was diminished in the cells treated with fasudil on the day 3 of differentiation (*P* < 0.001, Fig. 1h). However, fasudil had less effect on ATP production levels, which is known to be important for endoderm differentiation [25], than other ROCK inhibitors (*P* < *0.001*, Fig. S1c, d). Collectively, these results indicated that while fasudil transcriptionally affects by supplying more energy needed for differentiation in the early stages of hepatic differentiation of hUCM–MSCs compared to other ROCK inhibitors, but it does not influence next stage of endoderm due to organelle conditions.

### The effect of gelatin viscosity on hepatoblast induction of hUCM-MSCs

**Fig. 2:**
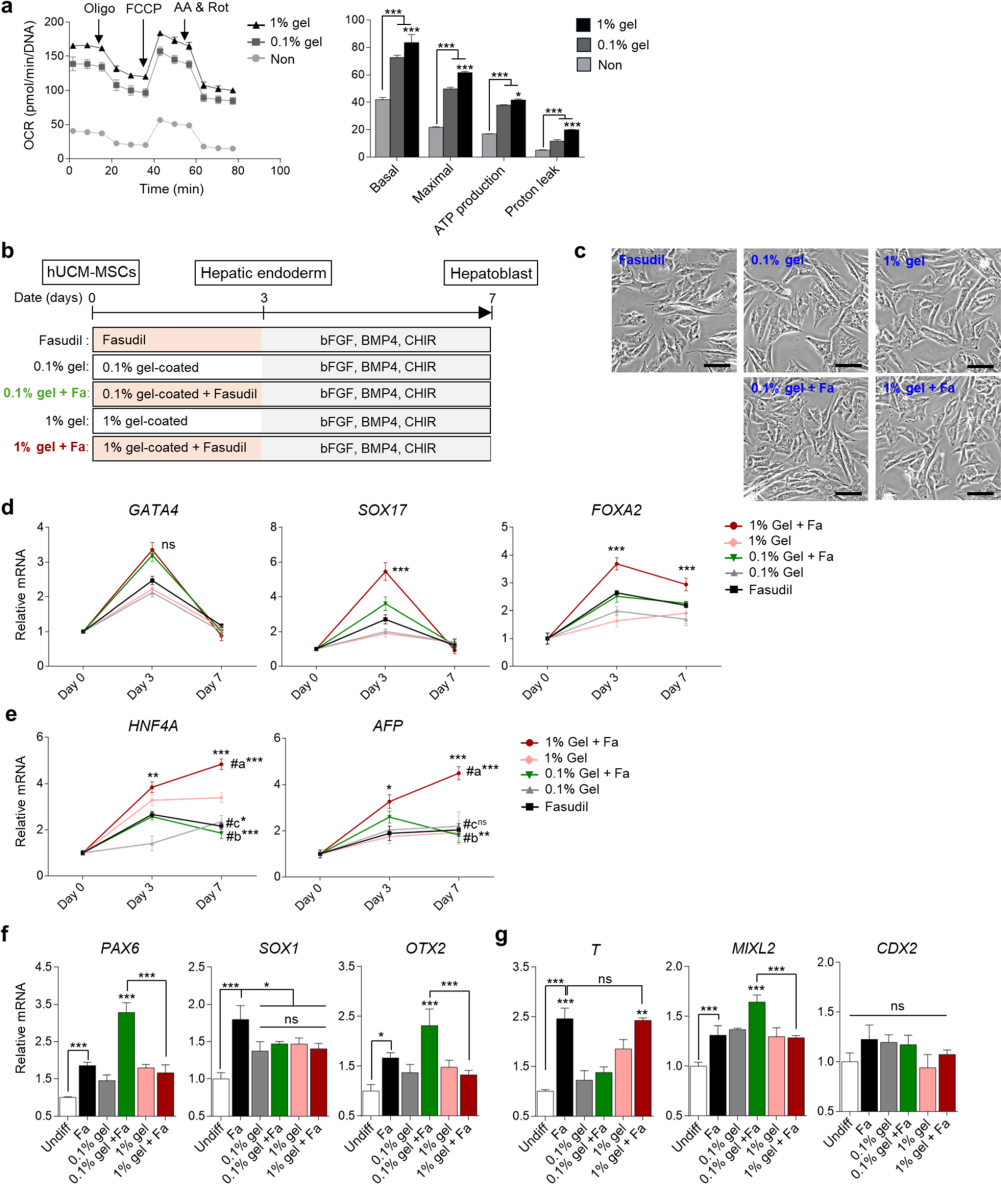
Transcription analysis of the effect of gelatin viscosity on early hepatic differentiation. **a** Time-dependent OCR graph and bar charts depending on gelatin-coating viscosity. OCR values were normalized by DNA concentration. **b** Schematic summary of early hepatic differentiation. Endoderm was induced depending on the experimental conditions for 3 days: Fasudil only, 0.1% gelatin-coated, 0.1% gelatin-coated and fasudil, 1% gelatin-coated, and 1% gelatin-coated and fasudil. And then, hepatoblasts were induced as indicated. CHIR: CHIR99021 **c** Morphology of the differentiated cells in each group on differentiation day 7. Scale bar = 100 μm. **d, e** Time-dependent RT-qPCR analysis of endoderm (d) and hepatoblast (e) markers on the differentiation day 0, 3, and 7. #a and #b are statistics comparing day 3 and 7 when 1% or 0.1% gelatin and fasudil were used. #c are statistics comparing day 3 and 7 when fasudil were only used. **f, g** Relative mRNA expression analysis of ectoderm (f) and mesoderm (g) markers for the differentiated cells on day 3. GAPDH was used as an internal control. *P* values < 0.05 were considered significant. *, *P* < 0.05; **, *P* < 0.01; ***, *P* < 0.001

Based on fasudil-treated results, we hypothesized that mitochondrial function and organelle conditions correlate with differentiation efficiency. Therefore, we aimed to enhance mitochondrial function and regulated organelle conditions through an extracellular matrix (ECM) component. Gelatin is known to low cost-biomaterial for stem cell culture and provides a suitable biological and differentiation signal for host cells [26–28]. Moreover, as confirmed in our result, when hUCM-MSCs were cultured on 0.1% or 1% gelatin-coated dishes, mitochondrial activation levels, such as basal, maximal oxidative phosphorylation, ATP production, and proton leak, were increased (*P* < 0.001, Fig. 2a). Gelatin also more improved the mitochondria function of hUCM-MSCs compared with other ECMSs used in stem cell research, such as Matrigel and vitronectin [29, 30] (Fig. S2a, b). Previous studies have shown that enhancement of oxidative phosphorylation levels and ATP production in differentiated cells is necessary for specific lineage differentiation [31, 32]. Therefore, we expected that gelatin used as ECM would synergistically improve the efficiency of differentiation with fasudil.

To assess gelatin’s impact on hepatic differentiation, we cultured hUCM-MSCs on 0.1% or 1% gelatin-coated dishes, and the differentiation proceeded under these conditions with fasudil (Fig. 2b). The phenotype of differentiated cells was similar on day 7 between all groups (Fig. 2c). Next, we analyzed the dynamics of gene expression associated with hepatic endoderm and hepatoblasts on the differentiation day 0, 3 and 7. As a result, endoderm-related genes (*SOX17* and *FOXA2*) except for *GATA4* and hepatoblast-related genes (*AFP* and *HNF4A*) exhibited significant upregulation in the 1% gelatin and fasudil group compared to other groups on day 3 and 7 (Fig. 2d, e and Fig. S4a, b). We also confirmed an influence of other ECMs on hepatic differentiation (Fig. S2c). The morphology of differentiated cells on differentiation day 7 were similar (Fig. S2d), but *SOX17*, *FOXA2* and *AFP* were more expressed on day 3 and 7 of differentiation when gelatin was used compared with other ECMs (Fig. S2e, f). However, interestingly, when hepatoblast differentiation was progressed, the hepatoblast markers (*AFP* and *HNF4A*) were decreased in the 0.1% gelatin and fasudil group, in contrast to the 1% gelatin and fasudil group (#a and #b, *P* < 0.01; Fig. 2e). Even when only fasudil was used, the hepatoblast genes were not increased (#c, *P* < 0.05; Fig. 2e). This result suggested that proper hepatic differentiation cannot be achieved with only fasudil or gelatin, and a high-viscosity gelatin coating is required for efficient differentiation using fasudil compared to low-viscosity coating.

To explain the variation in differentiation efficiency observed between the low-viscosity and high-viscosity gelatin groups, first, we examined the early differentiation fate of stem cells. Notably, fasudil has been documented as a promoter for the differentiation of cells originating from the ectoderm and mesoderm, including neurons and cardiomyocytes [33, 34]. Thus, we confirmed the gene expression of ectoderm (*PAX6*, *SOX1* and *OTX2*) and mesoderm (*T*, *MIXL2* and *CDX2*) markers in differentiated cells on day 3. As previously reported, when only fasudil was used, ectoderm- and mesoderm-related genes were upregulated except *CDX2* (*P* < 0.05, Fig. 2f, g). However, interestingly, the gene expression was different depending on gelatin viscosity. The expression of ectoderm genes (*PAX6* and *OTX2*) was suppressed in the 1% gelatin and fasudil group than 0.1% gelatin and fasudil-used group (*P* < 0.001; Fig. 2f). Furthermore, the mesoderm gene, *MIXL2*, was downregulated, but the mesendoderm gene, *T*, was upregulated in the 1% gelatin and fasudil group (*P* < 0.01; Fig. 2g). These results indicated that high-viscosity gelatin repressed the gene expression of ectoderm- and endoderm-related genes, thereby leading a more endodermal fate than low-viscosity gelatin.

Given that stem cell fate determinations according to gelatin viscosity can be influenced by the organelle states, next, we investigated the impact of high-viscosity gelatin on organelles in the differentiated cells.

### High-viscosity gelatin inhibited the induction of LDs and enhanced mitochondrial function

**Fig. 3:**
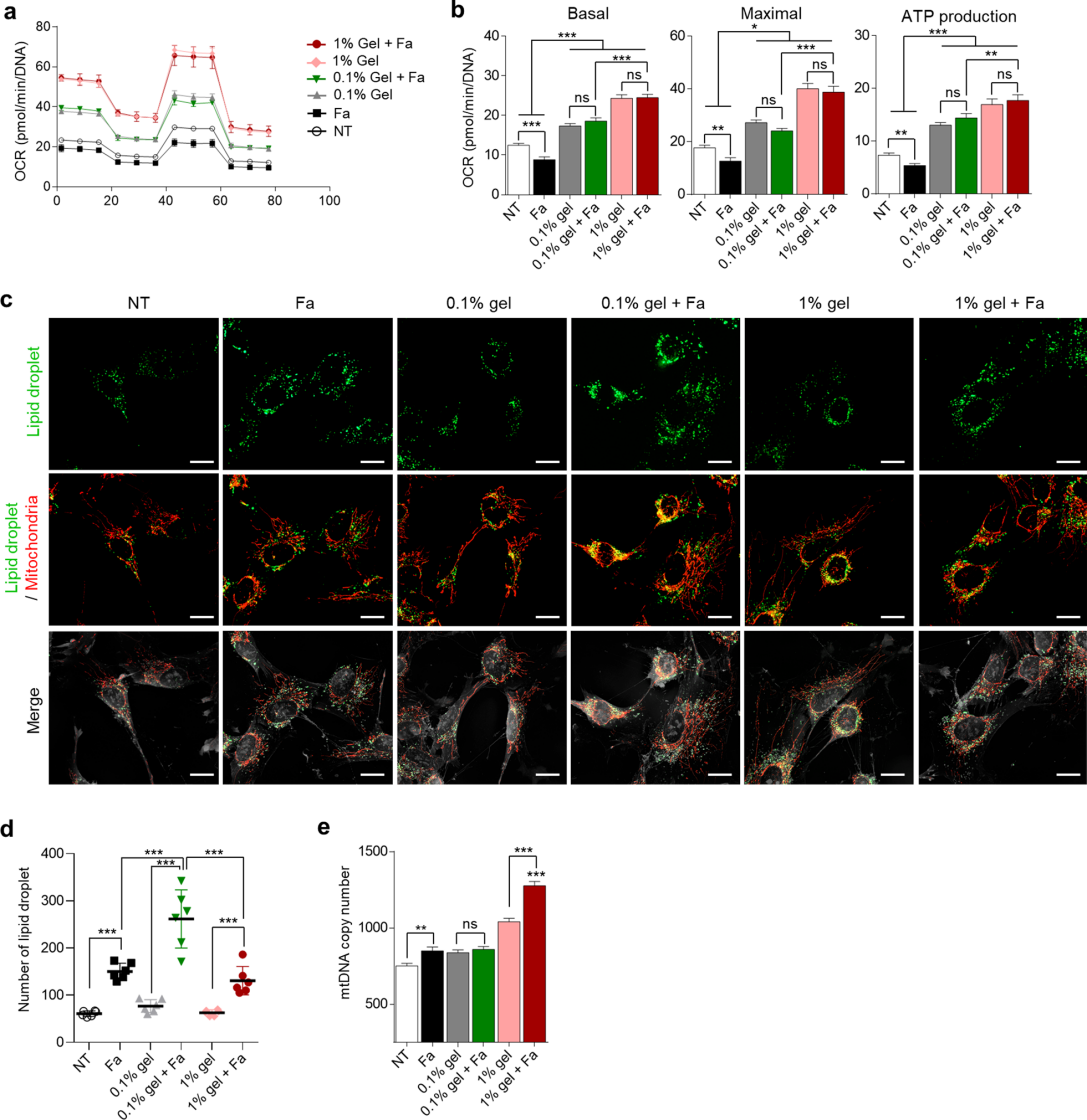
Analysis of lipid droplets and mitochondrial function in differentiated cells. **a** Time-dependent OCR levels for each group on differentiation day 3. **b** Bar charts showing the results of mitochondrial respiration changes in each group, analyzed with basal and maximal respiration, ATP production. OCR values were normalized by DNA concentration. **c** Tomographic analysis of the effect of gelatin and fasudil on hepatic differentiation. Mitochondria (red) and lipid droplets (green) were stained with MitoTracker and Biotium LipidSpot, respectively. Scale bar = 10 μm. **d** The number of lipid droplets in the cells of each group (each group, n = 6 cells). **e** Mitochondrial copy number analysis in the differentiated cells on day 3. *P* values < 0.05 were considered significant. *, *P* < 0.05; **, *P* < 0.01; ***, *P* < 0.001

To analyze the effects of high-viscosity gelatin on stem cell fate through regulation of organelles, mitochondrial function and tomographic analyses were performed. First, we conducted a seahorse assay to confirm whether gelatin increase mitochondrial functions of differentiated cells. As a result, mitochondria function increased depending on the viscosity of gelatin (*P* < 0.05, Fig. 3a, b). Furthermore, basal and maximal respiratory, and ATP production levels did not decrease when gelatin was used (Fig. 3b).

Next, we confirmed an expression of LDs through tomographic analysis to determine why the differentiated cells did not correctly differentiate into hepatoblasts despite restored mitochondrial function in low-viscosity gelatin. It is known that tomographic analysis can be used to observe cell organelles more accurately than confocal laser scanning microscopy [35]. Therefore, we tomographically analyzed LDs and morphology of the mitochondria in differentiated cells on day 3. The result showed that LDs were more accumulated in the 0.1% gelatin and fasduil-used group that only fasudil-used group, but the group treated with 1% gelatin and fasudil showed lower LDs induction than the group treated with 0.1% gelatin and fasudil (*P* < 0.001; Fig. 3c, d). Moreover, in the 0.1% gelatin and fasudil group, mitochondrial morphology appeared to be hyperfusion induced (Fig. 3c). This result suggested that low-viscosity gelatin upregulated mitochondrial function and promotes endoderm differentiation along with fasudil, but it accumulated excessive LDs within differentiated cells and hinders differentiation into hepatoblasts. On the other hand, high-viscosity gelatin greatly increased mitochondrial functions and inhibited the accumulation of excessive LDs within the cells, allowing hepatic differentiation to proceed appropriately. We also measured the mtDNA copy number across all groups. A previous study reported that mtDNA levels gradually increased to support differentiation [36]. Thus, we expected that the more efficient the differentiation, the higher the mtDNA copy number would be. In our study, the mtDNA copy number was higher in the 1% gelatin and fasudil group (*P* < 0.001, Fig. 3e).

In summary, the enhanced differentiation efficiency in the 1% gelatin and fasudil group can be attributed not only to the downregulation of ectodermal and mesodermal gene expression but also to the modulation of organelle states, including reduced LD production and activated mitochondrial function.

### Low-viscosity gelatin synergistically enhanced the efficiency and function of HLCs

**Fig. 4:**
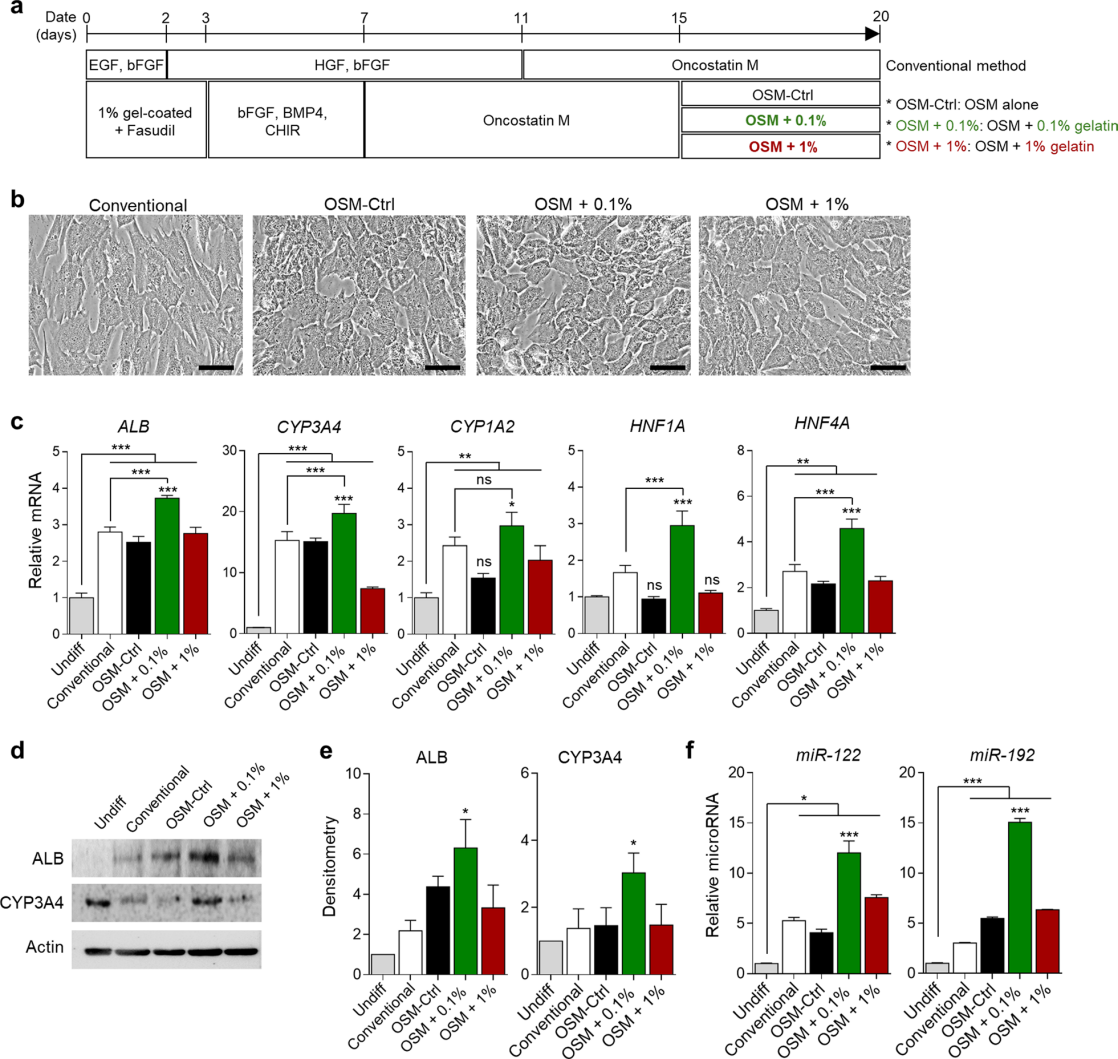
Low viscosity gelatin affected the maturation of hepatocyte-like cells from hUCM-MSCs. **a** The in vitro hepatic differentiation protocols using conventional method and 0.1% or 1% gelatin at the maturation stage. OSM: Oncostatin M. Conventional: Hepatocyte-like cells were induced by conventional method. CHIR: CHIR99021. **b** Morphology of the differentiated cells on day 20. Scale bar = 100 μm. **c** RT-qPCR analysis of mature hepatocyte markers on differentiation day 20. *GAPDH* was used as an internal control. **d** Western blotting for Albumin and CYP3A4 on the day 20-differentiated cells. Full-length blots are presented in Fig. S5. **e** Densitometry analysis of ALB and CYP3A4 (Biological replicate, n = 3). The densitometry values were normalized by actin. **f** Hepatic miR-122 and miR-192 transcript levels in the differentiated cells of each group at day 20. *RNU6B* was used as an internal control. *P* values < 0.05 were considered significant. *, *P* < 0.05; **, *P* < 0.01; ***, *P* < 0.001

Finally, we differentiated the hepatoblasts, which were induced using 1% gelatin coating and fasudil, into HLCs. At this stage, we used gelatin by adding step-2 differentiation medium rather than coating the dishes. Gelatin is known for its use in the long-term maintenance of human hepatocytes [37]. Thus, we assumed that it would help in the maturation of HLCs from hUCM-MSCs. To confirm the effect of gelatin on hepatocyte maturation, we induced HLCs from the hepatoblasts using OSM for 8 days, and we conducted the maturation by adding gelatin at different viscosities: 0% (no gelatin added) or 0.1% or 1% for 5 days. Moreover, an efficiency of the gelatin- and fasudil-used protocol was assessed compared with a differentiation method conventionally used for hUCM-MSC [19] (Fig. 4a). The HLC phenotypes were similar in all groups on differentiation day 20 (Fig. 4b). However, transcription analysis revealed that the expression of mature hepatocyte-related genes (*ALB, CYP3A4, CYP1A2, HNF1A* and *HNF4A*) was significantly elevated in the low-viscosity (0.1%) gelatin group compared to the others (*P* < 0.01, Fig. 4c). Moreover, the efficiency of differentiation improved compared to the conventional method except *CYP1A2* gene (*P* < 0.001, Fig. 4c). We also confirmed the expression of these proteins (Albumin; ALB and CYP3A4) by western blotting, wherein ALB and CYP3A4 levels in the 0.1% gelatin group was notably higher (*P* < 0.05, Fig. 4d, e). When comparing the efficiency of the differentiation method using low viscosity gelatin with the conventional method, ALB and CYP3A4 expression was also efficiently upregulated in the low-viscosity gelatin-used group (*P* < 0.05, Fig. 4d, e). We also assessed the maturation efficiency through the expression of hepatocyte-specific miRNAs, such as miR-122 and miR-192 [38]. This results also showed that hepatocyte-specific miRNAs expressed significantly upregulated in the presence of 0.1% gelatin (Fig. 4f). Furthermore, we also confirmed whether other ECMs can also affect hepatic maturation. Thus, two different ECMs, Matrigel and vitronectin, were added to the differentiation medium and the hepatic differentiation was conducted (Fig. S3a). As a result, the morphology of differentiated cells was similar (Fig. S3b), but the gene expression of *CYP3A4, CYP1A2* was higher in gelatin-used group than the other groups (*P* < 0.01, Fig. S3c). Moreover, consistent with the genetic results, the expression of CYP3A4 was higher in gelatin-used group compared to the other groups (*P* < 0.05, Fig. S3d, e). These results suggested that low viscosity of gelatin is helpful for hepatic differentiation and maturation than other expensive ECMs.

**Fig. 5:**
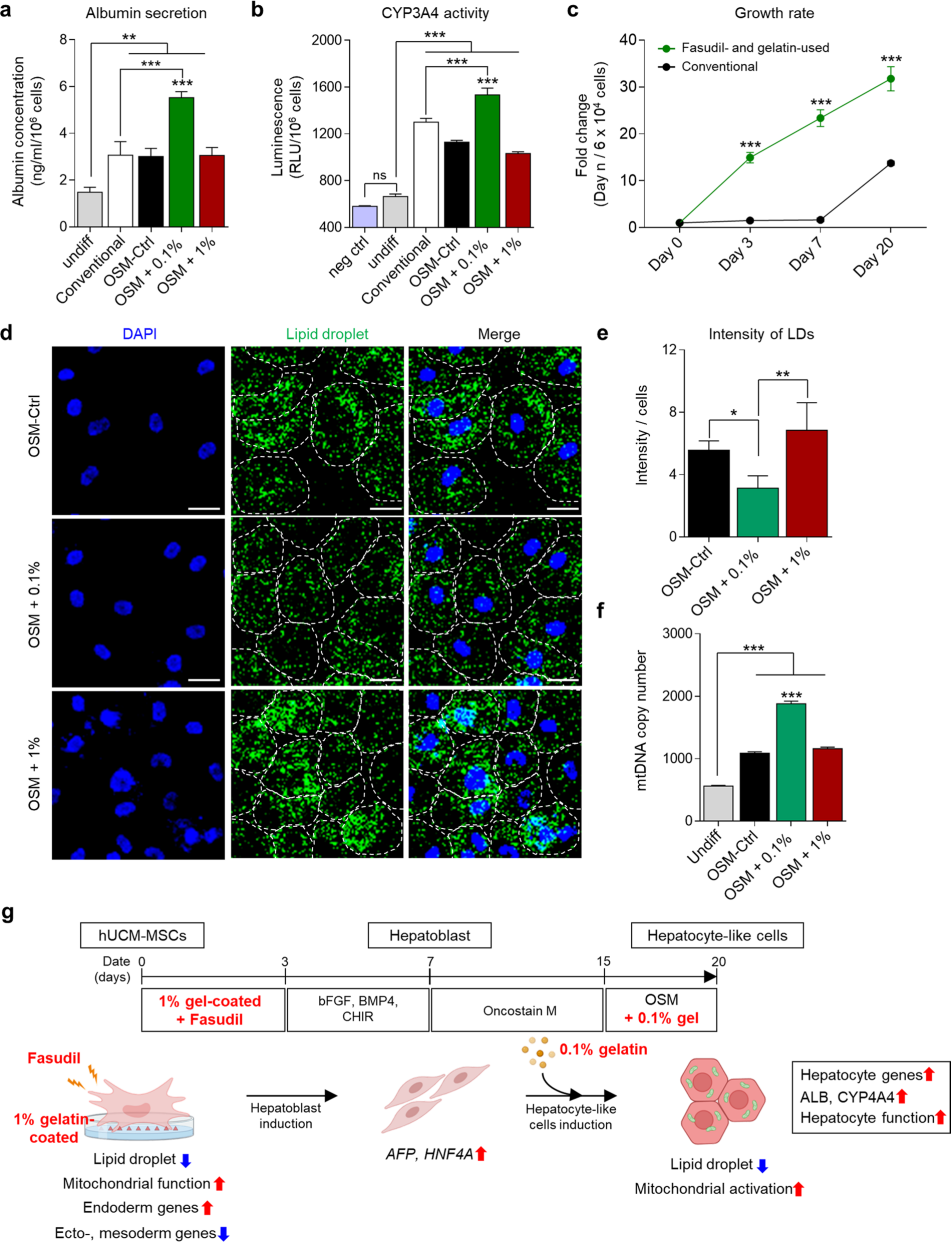
Viscosity of gelatin affected hepatic functions and organelles of mature hepatocyte-like cells. **a** Detection of human Albumin secretion in the differentiated cells on day 20, derived from each experimental group. Undiff, undifferentiated cells. Conventional: Hepatocyte-like cells were induced by conventional method. **b** Measurement of CYP3A4 activity using luminescence systems. Neg ctrl, only differentiation medium. **c** Comparison of growth rate between conventional protocol and gelatin- and fasudil-used protocol (Biological replication, n = 3). In the fasudil- and gelatin-used protocol, 1% gelatin coating and fasudil was used for endoderm induction, and 0.1% gelatin was used for hepatic maturation.** d** Immunocytochemical staining analysis of lipid droplets in the differentiated cells on day 20. Nuclei (blue) and lipid droplets (green) were stained with DAPI and Biotium LipidSpot, respectively. The boundaries of cells are indicated by white dotted lines. Scale bar = 20 μm. **e** Fluorescence intensity of detected LDs in (c) (n = 4). **f** Mitochondrial copy number analysis in the differentiated cells on day 20. **g** Schematic illustration of the overall flow of this study. *P* values < 0.05 were considered significant. *, *P* < 0.05; **, *P* < 0.01; ***, *P* < 0.001

Next, considering the primary roles of hepatocytes in protein synthesis and detoxification, we measured albumin secretion and CYP3A4 activity. The results showed that hepatic function and CYP3A4 activity were significantly upregulated in HLCs with the addition of 0.1% gelatin compared to other groups, including the conventional group (*P* < 0.001, Fig. 5a, b). We also counted the number of cells produced by the protocol using fasudil and gelatin to evaluate differentiation efficiency and compared with the conventional protocol. The number of differentiated cells was much higher than the conventional method (*P* < 0.001, Fig. 5c). Overall, the specification and maturation of hepatocytes were more efficient with low-viscosity gelatin. Therefore, we attempted to understand this phenomenon by analyzing the state of differentiated cell organelles, focusing on LDs and mtDNA copy number in the HLCs on day 20. Confocal imaging analysis confirmed that fewer LDs were induced when 0.1% gelatin was used for hepatocyte maturation (*P* < 0.05, Fig. 5d, e), and a significant increase in mtDNA copy number was observed in the same group (*P* < 0.001, Fig. 5f). These findings imply that low-viscosity gelatin is favorable for the maturation of HLCs, diverging from its role in early differentiation stages, and highlight that organelle states are pivotal in determining the efficiency and functionality of HLCs.

## Discussion

We successfully increased the efficiency of hepatic differentiation of hUCM-MSCs using fasudil and gelatin. Fasudil is a small molecule that is more stable and precise for stem cell differentiation than proteins used in the early stages of hepatic differentiation, such as activin A and Wnt3a [39]. Moreover, given that fasudil has been used in clinical treatment, it can be used in the production of stem cell-based therapies [40]. Thus, there is a necessary to discuss about the effects of fasudil on hUCM-MSCs. Previous studies have shown that fasudil prevents mitochondrial fission and induces fusion [25]. Consequently, it is possible that mitochondrial byproducts, such as reactive oxygen species—produced by fusion stress—change stem cell metabolism and increase differentiation-related genes [41]. For this reason, according to our findings, hepatoblast differentiation may have been hindered by mitochondrial stress. However, this assumption requires further investigation.

We also demonstrated that gelatin viscosity affects early and late hepatic differentiation of hUCM–MSCs. According to previous studies, the differentiation efficiency of human MSCs and liver stem cells increases in low-viscosity ECM, which provides soft stiffness [42, 43]. As in our study, the efficiency of hepatocyte maturation increased in low-viscosity gelatin in the late stages of differentiation. Contrary to the late stages, hepatoblast differentiation was enhanced on high-viscosity gelatin–coated dishes during the early stages. This means that the ECM requirement for differentiated cells varies depending on the developmental stage, and if not optimized, stem cell fate may change. Thus, our research suggests that to use ECM for differentiation, it is necessary to optimize the viscosity or concentration depending on the differentiation stage.

Next, we suggested that it may be important not only to induce appropriate transcription but also to induce the appropriate organelle state. The regulation of LDs and mitochondrial function had a significant impact on stem cell fate. Consistent with our findings, the condition of cellular organelles influences cellular metabolism, which can lead to epigenetic modifications [44]. Mitochondrial metabolism, glycolysis, and oxidative phosphorylation generate NADH/NAD^+^ and FADH_2_/FAD, which induce epigenetic changes such as histone methylation, acetylation, demethylation, and DNA demethylation [45]. This may serve as a crucial checkpoint for the therapeutic application of stem cell-derived differentiated cells.

Beyond the aspects already described, further detailed investigations are warranted. For example, the upstream factors that influence organelle behavior and transcriptional responses to fasudil and gelatin remain unidentified. Additionally, a comprehensive examination of organelles, including the Golgi apparatus and endoplasmic reticulum—both integral to cellular metabolism—is essential [46]. Establishing clear associations among organelle states, gene expression, and cellular functions in differentiated cells, and their comparison with human primary hepatocytes, could facilitate the therapeutic application of stem cell-derived hepatocytes in the future.

## Conclusion

In conclusion, we propose an effective protocol for hepatic differentiation using fasudil and gelatin. Fasudil prompted the early-stage induction of hUCM-MSCs into endoderm, while a high-viscosity (1%) gelatin coating modulates transcription and organelle states, thereby enhancing differentiation efficiency. Conversely, during the later stages of differentiation, a low gelatin viscosity (0.1%) enhanced hepatic maturation and function. At this stage, the suppression of lipid droplets and the activation of mitochondria also influenced maturation of HLCs (Fig. 5g). These results suggest that for the generation of differentiated cells suitable for cell therapy, a thorough analysis of organelle states, in addition to genetic and proteomic profiling, is crucial for the efficacy and functionality of hepatic cells differentiated from hUCM-MSCs.

### Supplementary Information

Additional file 1.

## Data Availability

The datasets used and/or analysed during the current study are available from the corresponding author on reasonable request.

## Abbreviations

MSCs: Mesenchymal stem cells
hUCM-MSCs: Human umbilical cord matrix-mesenchymal stem cells
HLCs: Hepatocyte-like cells
ROCK: Rho-associated protein kinase
LDs: Lipid droplets
mtDNA: Mitochondrial DNA
OSM: Oncostatin M
ODT: Label-free optical diffraction tomography
OCR: Oxygen consumption rate
ECM: Extracellular matrix
ATP: Adenosine triphosphate
ALB: Albumin
VTN: Vitronectin

## References

[1] Pittenger MF, Discher DE, Péault BM, Phinney DG, Hare JM, Caplan AI. Mesenchymal stem cell perspective: cell biology to clinical progress. npj Regenerative Medicine. 2019;4(1):22. 31815001 10.1038/s41536-019-0083-6 PMC6889290

[2] Ding D-C, Chang Y-H, Shyu W-C, Lin S-Z. Human umbilical cord mesenchymal stem cells: a new era for stem cell therapy. Cell Transp. 2015;24(3):339–47. 10.3727/096368915X686841 25622293

[3] Xie Q, Liu R, Jiang J, Peng J, Yang C, Zhang W, Wang S, Song J. What is the impact of human umbilical cord mesenchymal stem cell transplantation on clinical treatment? Stem Cell Res Ther. 2020;11(1):519. 33261658 10.1186/s13287-020-02011-z PMC7705855

[4] Rui LJCp. Energy metabolism in the liver. 2014;4(1):177. 10.1002/cphy.c130024 PMC4050641 24692138

[5] Jadlowiec CC, Taner TJ. Liver transplantation: current status and challenges. World J Gastroenterol. 2016;22(18):4438. 27182155 10.3748/wjg.v22.i18.4438 PMC4858627

[6] Stock P, Brückner S, Ebensing S, Hempel M, Dollinger MM, Christ B. The generation of hepatocytes from mesenchymal stem cells and engraftment into murine liver. Nat Protoc. 2010;5(4):617–27. 20224562 10.1038/nprot.2010.7

[7] Wnorowski A, Yang H, Wu JC. Progress, obstacles, and limitations in the use of stem cells in organ-on-a-chip models. Adv Drug Deliv Rev. 2019;140:3–11. 29885330 10.1016/j.addr.2018.06.001 PMC6281815

[8] Baxter M, Withey S, Harrison S, Segeritz CP, Zhang F, Atkinson-Dell R, Rowe C, Gerrard DT, Sison-Young R, Jenkins R, Henry J, Berry AA, Mohamet L, Best M, Fenwick SW, Malik H, Kitteringham NR, Goldring CE, Piper Hanley K, Vallier L, Hanley NA. Phenotypic and functional analyses show stem cell-derived hepatocyte-like cells better mimic fetal rather than adult hepatocytes. J Hepatol. 2015;62(3):581–9. 25457200 10.1016/j.jhep.2014.10.016 PMC4334496

[9] Afshari A, Shamdani S, Uzan G, Naserian S, Azarpira N. Different approaches for transformation of mesenchymal stem cells into hepatocyte-like cells. Stem Cell Res Ther. 2020;11(1):54. 32033595 10.1186/s13287-020-1555-8 PMC7007672

[10] Wells CA, Choi J. Transcriptional profiling of stem cells: moving from descriptive to predictive paradigms. Stem Cell Reports. 2019;13(2):237–46. 31412285 10.1016/j.stemcr.2019.07.008 PMC6700522

[11] Julian LM, Stanford WL. Organelle cooperation in stem cell fate: lysosomes as emerging regulators of cell identity. Fron Cell Dev Boil. 2020;8:591. 10.3389/fcell.2020.00591 PMC7358313 32733892

[12] Khacho M, Slack RS. Mitochondrial and reactive oxygen species signaling coordinate stem cell fate decisions and life long maintenance. Antioxid Redox Signal. 2018;28(11):1090–101. 28657337 10.1089/ars.2017.7228

[13] Kinney MA, Vo LT, Frame JM, Barragan J, Conway AJ, Li S, Wong KK, Collins JJ, Cahan P, North TE, Lauffenburger DA, Daley GQ. A systems biology pipeline identifies regulatory networks for stem cell engineering. Nat Biotechnol. 2019;37(7):810–8. 31267104 10.1038/s41587-019-0159-2 PMC7235931

[14] Intlekofer AM, Finley LWJ. Metabolic signatures of cancer cells and stem cells. Nat Metab. 2019;1(2):177–88. 31245788 10.1038/s42255-019-0032-0 PMC6594714

[15] Chen H, Chan DC. Mitochondrial dynamics in regulating the unique phenotypes of cancer and stem cells. Cell Metab. 2017;26(1):39–48. 28648983 10.1016/j.cmet.2017.05.016 PMC5539982

[16] Yue F, Oprescu SN, Qiu J, Gu L, Zhang L, Chen J, Narayanan N, Deng M, Kuang S. Lipid droplet dynamics regulate adult muscle stem cell fate. Cell Rep. 2022;38(3):110267. 35045287 10.1016/j.celrep.2021.110267 PMC9127130

[17] Lee J, Choi J, Kang S, Kim J, Lee R, So S, Yoon YI, Kirchner VA, Song GW, Hwang S, Lee SG, Kang E, Tak E. Hepatogenic potential and liver regeneration effect of human liver-derived mesenchymal-like stem cells. Cells. 2020;9(6):1521. 32580448 10.3390/cells9061521 PMC7348751

[18] Choi J, Kang S, Kim B, So S, Han J, Kim G-N, Lee M-Y, Roh S, Lee J-Y, Oh SJ, Sung YH, Lee Y, Kim SH, Kang E. Efficient hepatic differentiation and regeneration potential under xeno-free conditions using mass-producible amnion-derived mesenchymal stem cells. Stem Cell Res Ther. 2021;12(1):569. 34772451 10.1186/s13287-021-02470-y PMC8588618

[19] Campard D, Lysy PA, Najimi M, Sokal EM. Native umbilical cord matrix stem cells express hepatic markers and differentiate into hepatocyte-like cells. Gastroenterology. 2008;134(3):833–48. 18243183 10.1053/j.gastro.2007.12.024

[20] Maldonado M, Luu RJ, Ramos MEP, Nam J. ROCK inhibitor primes human induced pluripotent stem cells to selectively differentiate towards mesendodermal lineage via epithelial-mesenchymal transition-like modulation. Stem Cell Research. 2016;17(2):222–7. 27591478 10.1016/j.scr.2016.07.009

[21] Borowiak M, Maehr R, Chen S, Chen AE, Tang W, Fox JL, Schreiber SL, Melton DA. Small molecules efficiently direct endodermal differentiation of mouse and human embryonic stem cells. Cell Stem Cell. 2009;4(4):348–58. 19341624 10.1016/j.stem.2009.01.014 PMC4564293

[22] Korostylev A, Mahaddalkar PU, Keminer O, Hadian K, Schorpp K, Gribbon P, Lickert H. A high-content small molecule screen identifies novel inducers of definitive endoderm. Molecular metabolism. 2017;6(7):640–50. 28702321 10.1016/j.molmet.2017.04.009 PMC5485240

[23] Cui L, Liu P. Two types of contact between lipid droplets and mitochondria. Front Cell Dev Biol. 2020. 10.3389/fcell.2020.618322. 33385001 10.3389/fcell.2020.618322 PMC7769837

[24] Shares BH, Busch M, White N, Shum L, Eliseev RA. Active mitochondria support osteogenic differentiation by stimulating β-catenin acetylation. J Biol Chem. 2018;293(41):16019–27. 30150300 10.1074/jbc.RA118.004102 PMC6187642

[25] Lv J, Yi Y, Qi Y, Yan C, Jin W, Meng L, Zhang D, Jiang W. Mitochondrial homeostasis regulates definitive endoderm differentiation of human pluripotent stem cells. Cell Death Discov. 2022;8(1):69. 35177589 10.1038/s41420-022-00867-z PMC8854419

[26] Afewerki S, Sheikhi A, Kannan S, Ahadian S, Khademhosseini A. Gelatin-polysaccharide composite scaffolds for 3D cell culture and tissue engineering: Towards natural therapeutics. Bioeng Transl Med. 2019;4(1):96–115. 30680322 10.1002/btm2.10124 PMC6336672

[27] Chhabra H, Gupta P, Verma PJ, Jadhav S, Bellare JR. Gelatin–PMVE/MA composite scaffold promotes expansion of embryonic stem cells. Mater Sci Eng C. 2014;37:184–94. 10.1016/j.msec.2013.12.033 24582239

[28] Arkenberg MR, Koehler K, Lin C-C. Heparinized gelatin-based hydrogels for differentiation of induced pluripotent stem cells. Biomacromol. 2022;23(10):4141–52. 10.1021/acs.biomac.2c00585 PMC9554908 36074748

[29] Novoseletskaya E, Grigorieva O, Nimiritsky P, Basalova N, Eremichev R, Milovskaya I, Kulebyakin K, Kulebyakina M, Rodionov S, Omelyanenko N, Efimenko A. Mesenchymal stromal cell-produced components of extracellular matrix potentiate multipotent stem cell response to differentiation stimuli. Front Cell Dev Biol. 2020. 10.3389/fcell.2020.555378. 33072743 10.3389/fcell.2020.555378 PMC7536557

[30] Zhou Y, Zhou J, Xu X, Du F, Nie M, Hu L, Ma Y, Liu M, Yu S, Zhang J, Chen Y. Matrigel/umbilical cord-derived mesenchymal stem cells promote granulosa cell proliferation and ovarian vascularization in a mouse model of premature ovarian failure. Stem Cells Dev. 2021;30(15):782–96. 34030464 10.1089/scd.2021.0005

[31] Cho YM, Kwon S, Pak YK, Seol HW, Choi YM, Park DJ, Park KS, Lee HKJB. Dynamic changes in mitochondrial biogenesis and antioxidant enzymes during the spontaneous differentiation of human embryonic stem cells. Biochem Biophys Res Commun. 2006;348(4):1472–8. 16920071 10.1016/j.bbrc.2006.08.020

[32] Chung S, Arrell DK, Faustino RS, Terzic A, Dzeja PP. Glycolytic network restructuring integral to the energetics of embryonic stem cell cardiac differentiation. J Mol Cell Cardiol. 2010;48(4):725–34. 20045004 10.1016/j.yjmcc.2009.12.014 PMC2837789

[33] Khan AA, Huat TJ, Al Mutery A, El-Serafi AT, Kacem HH, Abdallah SH, Reza MF, Abdullah JM, Jaafar H. Significant transcriptomic changes are associated with differentiation of bone marrow-derived mesenchymal stem cells into neural progenitor-like cells in the presence of bFGF and EGF. Cell Biosci. 2020;10(1):126. 33133516 10.1186/s13578-020-00487-z PMC7594431

[34] Hu Y, Li X, Huang G, Wang J, Lu W. Fasudil may induce the differentiation of bone marrow mesenchymal stem cells into neuron-like cells via the Wnt/β-catenin pathway. Mol Med Rep. 2019;19(4):3095–104. 30816472 10.3892/mmr.2019.9978 PMC6423592

[35] Kim Y, Kim T-K, Shin Y, Tak E, Song G-W, Oh Y-M, Kim JK, Pack C-G. Characterizing organelles in live stem cells using label-free optical diffraction tomography. Mol Cells. 2021;44(11):851–60. 34819398 10.14348/molcells.2021.0190 PMC8627838

[36] Hu C, Fan L, Cen P, Chen E, Jiang Z, Li L. Energy metabolism plays a critical role in stem cell maintenance and differentiation. Int J Mol Sci. 2016;17(2):253. 26901195 10.3390/ijms17020253 PMC4783982

[37] Klaas M, Möll K, Mäemets-Allas K, Loog M, Järvekülg M, Jaks V. Long-term maintenance of functional primary human hepatocytes in 3D gelatin matrices produced by solution blow spinning. Sci Rep. 2021;11(1):20165. 34635750 10.1038/s41598-021-99659-1 PMC8505433

[38] Xiaolin W, Yong H, Bryan M, Bin G. MicroRNAs as regulators, biomarkers and therapeutic targets in liver diseases. Gut. 2021;70(4):784. 33127832 10.1136/gutjnl-2020-322526

[39] Pavathuparambil Abdul Manaph N, Sivanathan KN, Nitschke J, Zhou X-F, Coates PT, Drogemuller CJ. An overview on small molecule-induced differentiation of mesenchymal stem cells into beta cells for diabetic therapy. Stem Cell Res Ther. 2019;10(1):293. 31547868 10.1186/s13287-019-1396-5 PMC6757413

[40] Feng Y, LoGrasso PV, Defert O, Li R. Rho kinase (ROCK) inhibitors and their therapeutic potential. J Med Chem. 2016;59(6):2269–300. 26486225 10.1021/acs.jmedchem.5b00683

[41] Xiao F, Zhang R, Wang L. Inhibitors of mitochondrial dynamics mediated by dynamin-related protein 1 in pulmonary arterial hypertension. Front Cell Dev Biol. 2022. 10.3389/fcell.2022.913904. 35846374 10.3389/fcell.2022.913904 PMC9280643

[42] Lee K, Chen Y, Yoshitomi T, Kawazoe N, Yang Y, Chen G. Osteogenic and adipogenic differentiation of mesenchymal stem cells in gelatin solutions of different viscosities. Adv Healthcare Mater. 2020;9(23):2000617. 10.1002/adhm.202000617 32755043

[43] Cozzolino AM, Noce V, Battistelli C, Marchetti A, Grassi G, Cicchini C, Tripodi M, Amicone L. Modulating the substrate stiffness to manipulate differentiation of resident liver stem cells and to improve the differentiation state of hepatocytes. Stem Cells Int. 2016;2016:5481493. 27057172 10.1155/2016/5481493 PMC4737459

[44] Dai Z, Ramesh V, Locasale JW. The evolving metabolic landscape of chromatin biology and epigenetics. Nat Rev Genet. 2020;21(12):737–53. 32908249 10.1038/s41576-020-0270-8 PMC8059378

[45] Tatapudy S, Aloisio F, Barber D, Nystul T. Cell fate decisions: emerging roles for metabolic signals and cell morphology. EMBO Rep. 2017;18(12):2105–18. 29158350 10.15252/embr.201744816 PMC5709733

[46] Sekine Y, Houston R, Sekine S. Cellular metabolic stress responses via organelles. Exp Cell Res. 2021;400(1): 112515. 33582095 10.1016/j.yexcr.2021.112515

