# Supplementary material for: Fasudil and viscosity of gelatin promote hepatic differentiation by regulating organelles in human umbilical cord matrix-mesenchymal stem cells
Source: Stem Cell Res Ther. 2024 Jul 29;15:229. doi: 10.1186/s13287-024-03851-9 (PMC11288082; doi:10.1186/s13287-024-03851-9)
Supplement: Supplementary file 1 — Additional file 1. [file 13287_2024_3851_MOESM1_ESM.pdf]

Table S1. primer list

Gene name	Sequence Forward	Reverse
GATA4	GGCCTGTCATCTCACTACGG	ATGGCCAGACATCGCACT
SOX17	CAAGGGCGAGTCCCGTAT	CGACTTGCCCAGCATCTT
FOXA2	GGAGCGGTGAAGATGGA	TCATGTTGCTCACGGAGGA
PAX6	GAGTTCTTCGCAACCTGGCTA	CTGCCCGTTCAACATCCTTAG
SOX1	AGTGGAAGGTCATGTCCGA	TTCTTGAGCAGCGTCTTGGT
OTX2	AGAGCTAAGTGCCGCCAAC	TCCGAGCTGGAGATGTCTT
T	GGTCCACAGCGCATGATC	TGATAAGCAGTCACCGCTATGAA
MIXL2	TTTGGCTAGGCCGGAGATTA	GCAGGCAGTTCACATCTACCT
CDX2	ACAGTCGCTACATCACCATCC	CTCTCCTTTGCTCTGCGGTT
AFP	AGACTGCTGCAGCCAAAGTGA	GTGGGATCGATGCTGGAGTG
HNF4A	CAGGCTCAAGAAATGCTTCC	GGCTGCTGTCCTCATAGCTT
ALBUMIN	CACAGAATCCTTGGRGAACAGG	ATGGAAGGTGAATGTTTCAGCA
CYP3A4	TTTTGTCCTACCATAAGGGCTTT	CACAGGCTGTTGACCATCAT
CYP1A2	GGACAGCACTTCCCTGAGA	AGGCAGGTAGCGAAGGATG
HNF1A	TGGGTCCTACGTTCACCAAC	TCTGCACAGGTGGCATGAG

Fig. S1

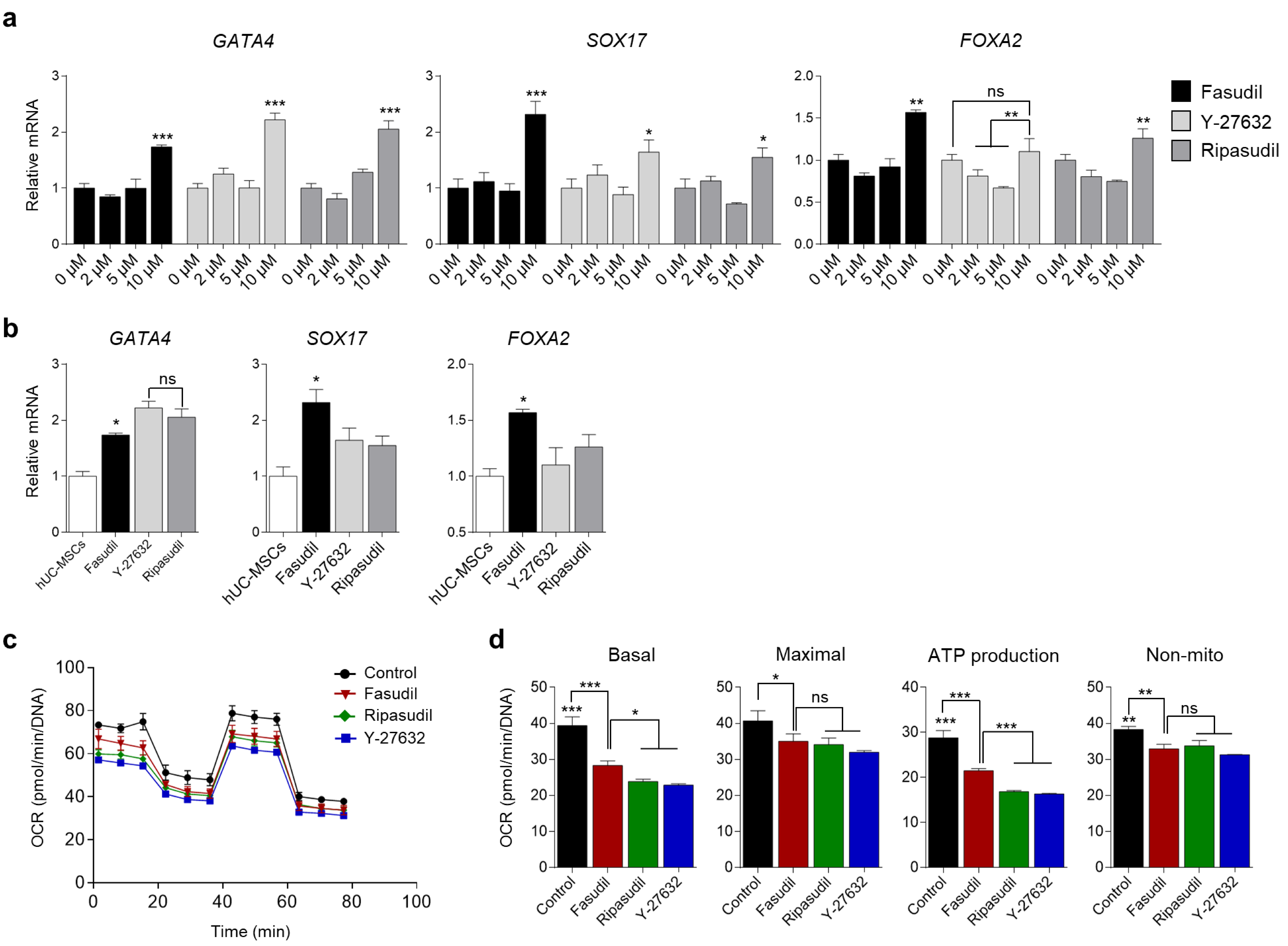

Figure S1. Effect of three different ROCK inhibitors on early hepatic differentiation of hUCM-MSCs

a Relative mRNA expression analysis of endodermal markers (GATA4, SOX17, and FOXA2) in the cells treated with each ROCK inhibitors according to concentration. **b** Comparison of endodermal marker's mRNA expression levels when each ROCK inhibitor was treated with 10 μm concentration for 3 days. GAPDH was used as an internal control. **c, d** Time-dependent OCR level graph (c) and bar charts (d) depending on three different ROCK inhibitors. Each inhibitor was treated at a concentration of 10 μm for 3 days. OCR values were normalized by DNA concentration. P values < 0.05 were considered significant. *, P < 0.05; **, P < 0.01; ***, P < 0.001.

Fig. S2

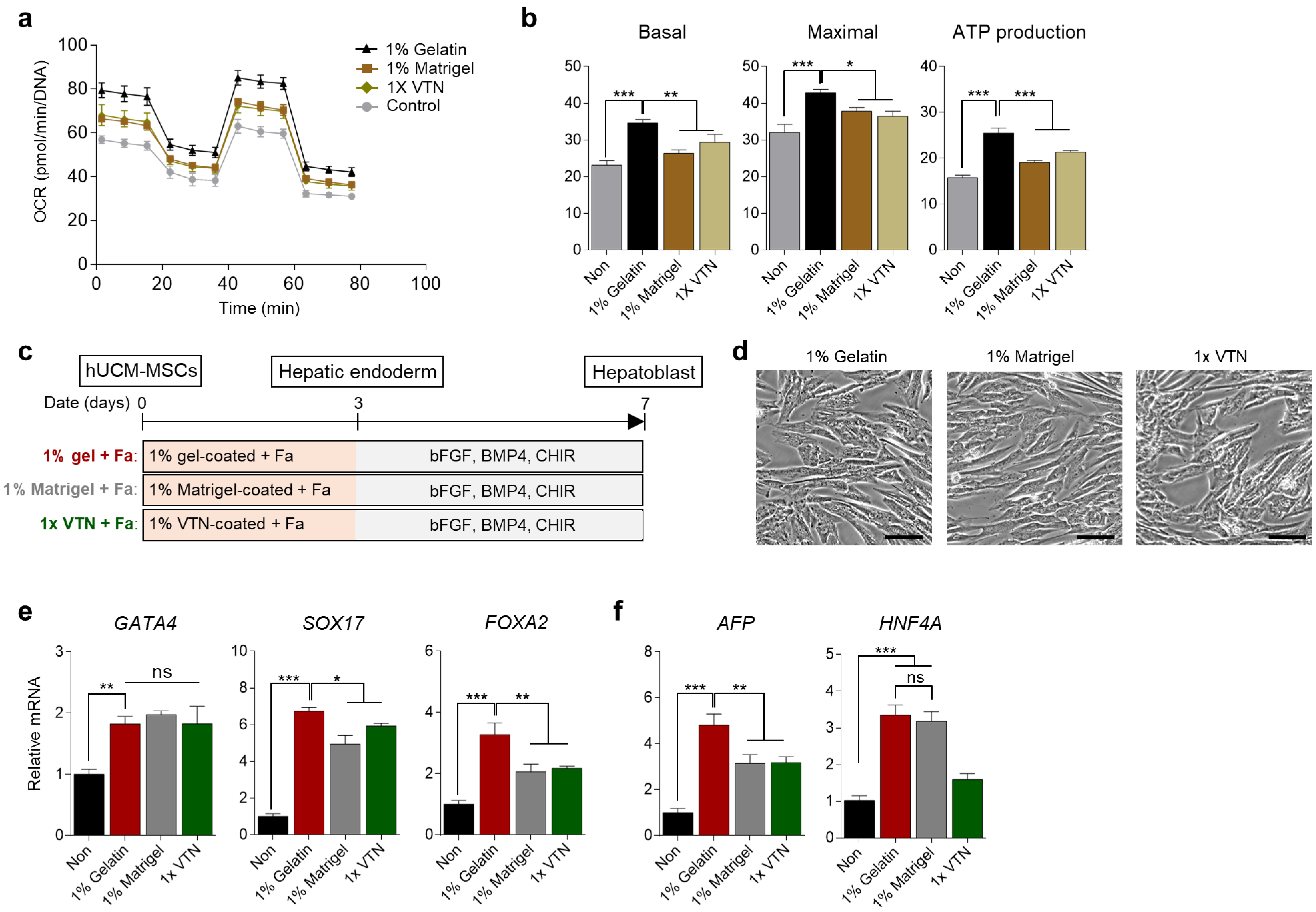

Figure S2. Effect of three different extracellular matrix on early hepatic differentiation of hUCM-MSCs

a, b Time-dependent OCR level graph (a) and bar charts (b) of mitochondria function depending on three different ECMs. OCR values were normalized by DNA concentration. **c** Schematic summary of hepatic differentiation on three different ECMs-coated dishes with fasudil. **d** Morphology of the differentiated cells on differentiation day 7. Scale bar = 100 µm. **e, f** RT-qPCR analysis of endoderm (e) markers on differentiation day 3 and hepatoblast (f) markers on differentiation day 7. GAPDH was used as an internal control. P values < 0.05 were considered significant. *, $P < 0.05$; **, $P < 0.01$; ***, $P < 0.001$.

Fig. S3

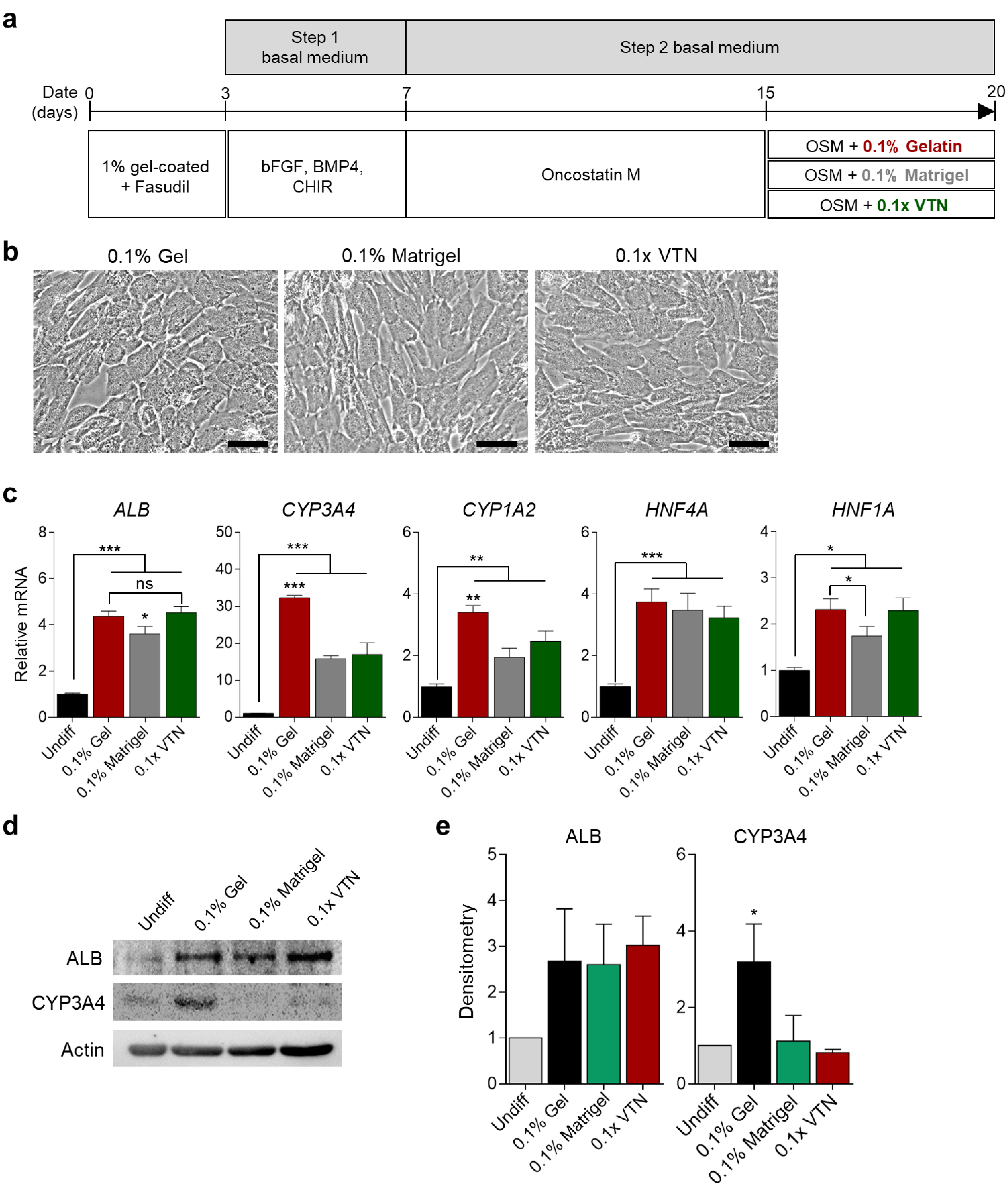

Figure S3. Analysis of the impact of three different extracellular matrix on differentiated hepatocyte-like cell's maturation

a The in vitro hepatic differentiation protocols using 0.1% gelatin or 0.1% Matrigel or 0.1x VTN at the maturation stage. CHIR: CHIR99021. **b** Morphology of the differentiated cells on day 20 depending on the use of three different ECMs as additives. Scale bar = 100 μm. **c** Transcription analysis of mature hepatocyte markers on differentiation day 20. GAPDH was used as an internal control. **d** Western blotting for Albumin and CYP3A4 on the day 20-differentiated cells. The maturation was induced by three different ECMs. Full-length blots are presented in Figure S5. **e** Densitometry analysis of ALB and CYP3A4 (Biological replicate, n = 3). The densitometry values were normalized by actin. P values < 0.05 were considered significant. *, $P < 0.05$; **, $P < 0.01$; ***, $P < 0.001$.

Fig. S4

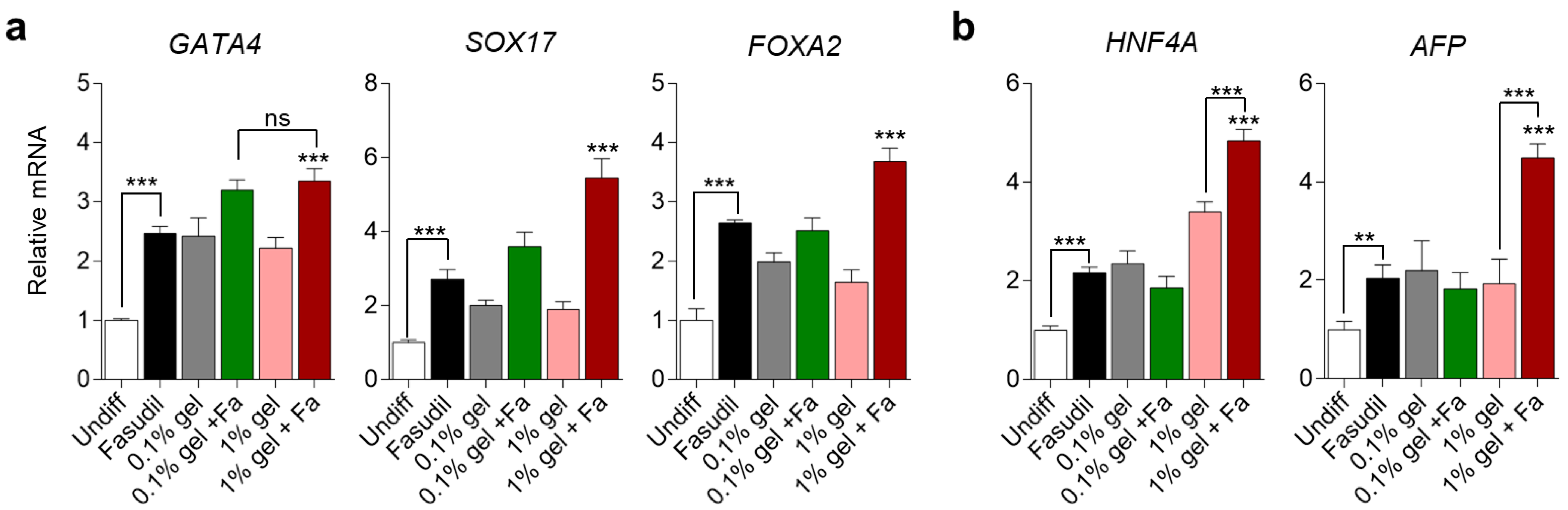

Figure S4. Transcription analysis of the effect of gelatin viscosity on early hepatic differentiation day 3 and 7

a Bar graph of RT-qPCR analysis of endoderm markers on the differentiation day 3 in Fig. 2d. **b** Bar graph of RT-qPCR analysis of hepatoblast markers on the differentiation day 7 in Fig. 2e. GAPDH was used as an internal control. P values < 0.05 were considered significant. *, P < 0.05; **, P < 0.01; ***, P < 0.001.

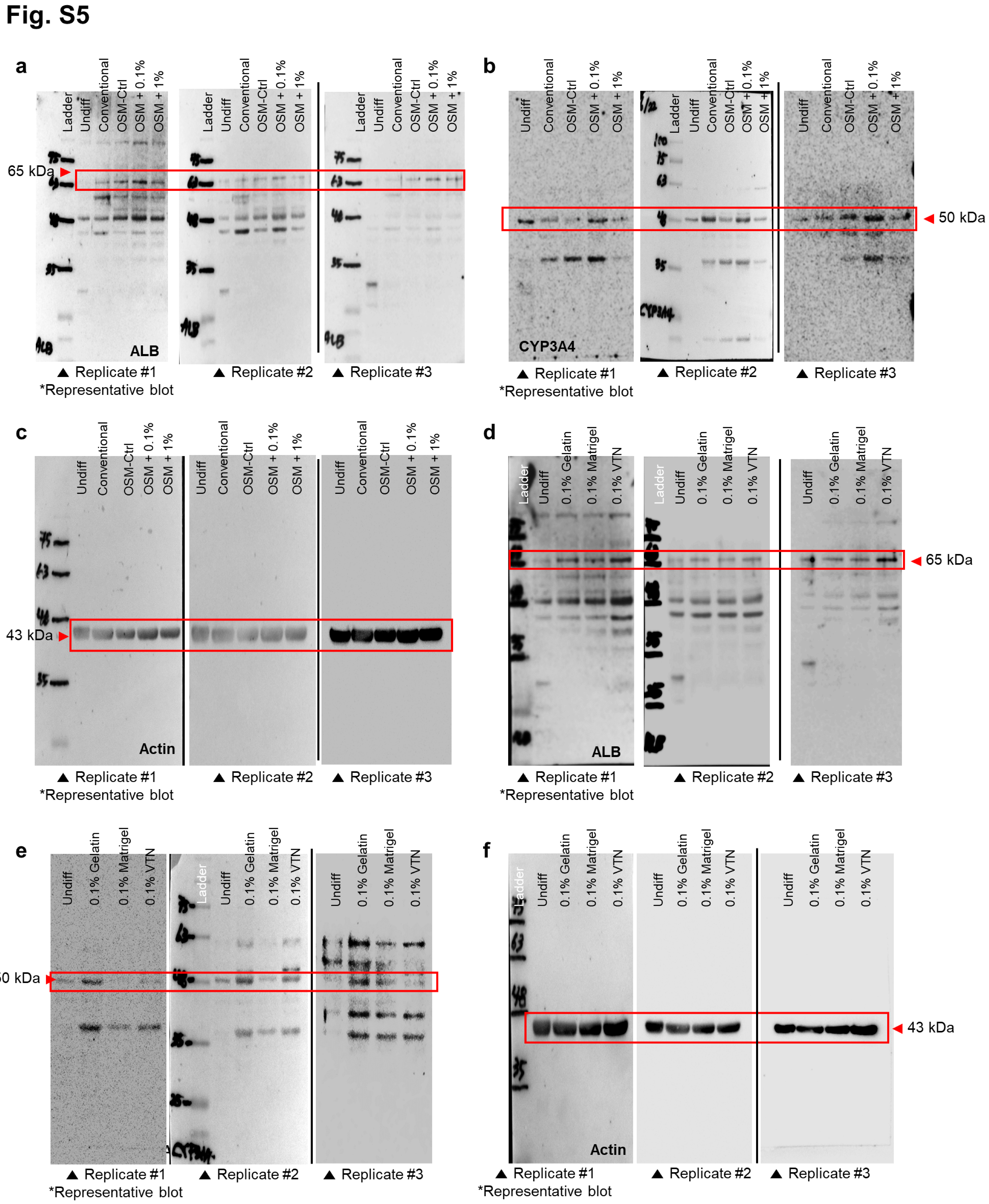

Figure S5. Uncropped full-length of Western blot membrane

a, b, c Whole membrane of Western blotting of human Albumin (a), CYP3A4 (b), and Actin (c) of Fig. 4d.

d, e, f Whole membrane of Western blotting of human Albumin (d), CYP3A4 (e), and Actin (f) of Fig. S3d
